# Older African Americans with Type 2 Diabetes have Lower Frontal and Medial Temporal Lobe Cognitive Function

**DOI:** 10.1002/alz.091473

**Published:** 2025-01-03

**Authors:** Darian A. Napoleon, Bernadette A. Fausto, Wiktoria Piaszczynska, Fanyu Hercules Tawe, Miray Budak, Steven K. Malin, Patricia Fitzgerald‐Bocarsly, Mark A. Gluck

**Affiliations:** ^1^ Rutgers University–Newark, Newark, NJ USA; ^2^ Rutgers, The State University of New Jersey, New Brunswick, NJ USA; ^3^ Rutgers New Jersey Medical School, Newark, NJ USA

## Abstract

**Background:**

Alzheimer’s disease (AD) is sometimes characterized as “type 3 diabetes” because hyperglycemia impairs cognitive function, particularly in the medial temporal lobe (MTL) and prefrontal regions^1,2^. Further, both AD and type 2 diabetes (T2D) disproportionately impact African Americans. Although people with T2D are generally suggested to have lower episodic memory and executive function, limited data exist in older African Americans. Thus, we examined the effect of T2D on frontal and MTL cognitive functions in older African Americans.

**Method:**

Eighty‐four older cognitively unimpaired African Americans (70.88±6.31y; 74% female) were recruited from the *Pathways to Healthy Aging in African Americans* study at Rutgers University–Newark. Individuals were categorized as having T2D using HbA1c (≥6.5%; *n* = 48) or non‐T2D (≤6.4%; *n* = 36). Participants completed a cognitive battery of frontal and MTL cognitive tasks: motor speed of processing (Trail Making Test–Part A); set‐shifting (Trail Making Test–Part B); and generalization of prior learning (Concurrent Discrimination and Transfer Task). ANCOVAs were used to examine differences between groups on cognitive performance with age, sex, and education as covariates.

**Result:**

Participants with T2D demonstrated poorer motor speed of processing (*p* = 0.046, η^2^_p_ = 0.05) and set‐shifting performance (*p* = 0.05; η^2^_p_ = 0.047) than those without T2D. Moreover, participants with T2D demonstrated lower generalization of prior learning accuracy (*p* = 0.05; η^2^_p_ = 0.047).

**Conclusion:**

T2D lowers frontal and MTL function in cognitively unimpaired older African Americans with when compared to those without diabetes. Further investigation into the overlapping pathologies of AD and T2D are warranted to optimize interventions aimed at prevention of AD in older adults.

